# The biological function of β-secretase

**DOI:** 10.1186/1750-1326-8-S1-O5

**Published:** 2013-09-13

**Authors:** Christian Haass

**Affiliations:** 1 Adolf Butenandt-Institute, Biochemistry, Ludwig-Maximilians Universität München and German Center for Neurodegenerative Diseases (DZNE), Germany

## 

Proteolytic shedding of cell surface proteins generates paracrine signals involved in numerous signaling pathways. Neuregulin 1 (NRG1) type III is involved in myelination of the peripheral nervous system, for which it requires proteolytic activation by proteases of the ADAM family and BACE1. These proteases are major therapeutic targets for the prevention of Alzheimer’s disease because they are also involved in the proteolytic generation of the neurotoxic amyloid β-peptide. Identification and functional investigation of their physiological substrates is therefore of greatest importance in preventing unwanted side effects. Here we investigated proteolytic processing of NRG1 type III and demonstrate that the ectodomain can be cleaved by three different sheddases, namely ADAM10, ADAM17, and BACE1. Surprisingly, we not only found cleavage by ADAM10, ADAM17, and BACE1 C-terminal to the epidermal growth factor (EGF)-like domain, which is believed to play a pivotal role in signaling, but also additional cleavage sites for ADAM17 and BACE1 N-terminal to that domain. Proteolytic processing at N- and C-terminal sites of the EGF-like domain results in the secretion of this domain from NRG1 type III. The soluble EGF-like domain is functionally active and stimulates ErbB3 signaling in tissue culture assays. Moreover, the soluble EGF-like domain is capable of rescuing hypomyelination in a zebrafish mutant lacking BACE1. Our data suggest that NRG1 type III independent myelination is not only controlled by membrane-retained NRG1 type III, but also in a paracrine manner via proteolytic liberation of the EGF-like domain.

